# Supplementary material for: De novo design of buttressed loops for sculpting protein functions
Source: Nat Chem Biol. 2024 May 30;20(8):974–80. doi: 10.1038/s41589-024-01632-2 (PMC11288887; doi:10.1038/s41589-024-01632-2)
Supplement: Supplementary file 1 — Supplementary Table 1. [file 41589_2024_1632_MOESM1_ESM.pdf]

nature chemical biology

Supplementary information

https://doi.org/10.1038/s41589-024-01632-2

De novo design of buttressed loops for sculpting protein functions

In the format provided by the authors and unedited

Supplementary Table 1. Crystallographic data collection and refinement statistics.

	RBL4	RBL7_C2_3
**Wavelength (Å)**	1.033	0.9795
**Resolution range (Å)**	59.84 - 1.8 (1.864 - 1.8)	45.3 - 2.986 (3.092 - 2.986)
**Space group**	P 21 21 2	P 21 21 21
**Unit cell (Å, °)**	62.287 215.442 28.008 90 90 90	111.961 117.644 142.056 90 90 90
**Total reflections**	258923 (25447)	258197 (25212)
**Unique reflections**	35977 (3521)	38693 (3751)
**Multiplicity**	7.2 (7.2)	6.7 (6.7)
**Completeness (%)**	98.67 (96.09)	99.02 (92.46)
**Mean I/sigma(I)**	5.70 (0.36)	5.34 (0.64)
**Wilson B-factor**	40.77	67.39

**R-sym**	0.1381 (3.305)	0.3314 (3.08)
**R-meas**	0.1498 (3.557)	0.3597 (3.336)
**R-pim**	0.05685 (1.298)	0.1385 (1.269)
**CC1/2**	0.989 (0.335)	0.996 (0.395)
**Reflections used in refinement**	35803 (3439)	38424 (3544)
**Reflections used for R-free**	1974 (193)	1988 (179)
**R-work**	0.2329 (0.6169)	0.2097 (0.3431)
**R-free**	0.2835 (0.6811)	0.2593 (0.3912)
**Number of non-hydrogen atoms**	3216	12376
**macromolecules**	3066	12331
**ligands**	28	7
**solvent**	122	38

**Protein residues**	410	1648
**RMS(bonds) (Å)**	0.011	0.004
**RMS(angles) (°)**	1.07	0.70
**Ramachandran favored (%)**	99.51	98.35
**Ramachandran allowed (%)**	0.49	1.65
**Ramachandran outliers (%)**	0.00	0.00
**Rotamer outliers (%)**	0.96	3.80
**Clashscore**	2.22	7.38
**Average B-factor (Å^2)**	52.05	77.82
**macromolecules**	52.07	77.86
**ligands**	51.18	121.17
**solvent**	51.77	55.70

**Number of TLS groups**	4	8

Statistics for the highest-resolution shell are shown in parentheses.
